# A Pathfinder in High-Pressure Bioscience: In Memoriam of Gaston Hui Bon Hoa

**DOI:** 10.3390/biology10080778

**Published:** 2021-08-16

**Authors:** Dmitri R. Davydov, Christiane Jung, Gregory A. Petsko, Stephen G. Sligar, Jack A. Kornblatt

**Affiliations:** 1 Department of Chemistry, Washington State University, Pullman, CA 99164-4630, USA; 2 KKS Ultraschall AG, Medical Surface Center, 6422 Steinen, Switzerland; 3 Ann Romney Center for Neurologic Diseases, Department of Neurology, Harvard Medical School and Brigham & Women’s Hospital, Boston, MA 02115, USA; gpetsko@bwh.harvard.edu; 4 Department of Chemistry, University of Illinois, Urbana, IL 61801, USA; s-sligar@illinois.edu; 5 Department of Biology, Concordia University, Montréal, QC H4B 1R6, Canada; Jack.Kornblatt@concordia.ca

On 26 July 2020, our colleague and friend Dr. Gaston Hui Bon Hoa passed away. Gaston was an enthusiastic scientist. He was one of the pioneers studying the effects of hydrostatic pressure on proteins, nucleic acids, and their assemblies. He devoted over 40 years of his scientific career to establishing pressure perturbation approaches and applying them in the study of protein structure and function. We dedicate this Special Issue to the memory of Gaston Hui Bon Hoa.

Gaston Hui Bon Hoa was born on 13 May 1934 in Ku Lang Su, China. Gaston’s family originated from the Chinese Fujian province but had French citizenship since 1887 and resided in Vietnam. By the end of the French presence in Indochina, most of his family members had migrated to France. Gaston Hui Bon Hoa arrived in Paris with his parents and brother in August 1951. Here, he attended a state school. In 1956 Gaston enrolled at the Department of Physics at the Faculty of Science, Paris-Sorbonne University. In 1962, he received his license (B.S.) diploma in physics and electronics from the University Paris XI (Orsay).

Most of Gaston’s scientific carrier was associated with the Institute of Physical-Chemical Biology (Institut de Biologie Physico-Chimique, IBPC, Paris, France). In 1965, he joined the Department of Bio-Spectroscopy at IBPC, headed by Professor Pierre Douzou, as a Research Fellow. In 1974 Gaston obtained his Ph.D. degree from the University Paris XI for the study “Establishing experimental conditions for low-temperature studies of proteins” supervised by Professor Pierre Douzou. Gaston continued his carrier under Prof. Douzou’s mentorship as a permanent staff member of the National Institute of Health and Medical Research (INSERM, Unité 310) (Figure 1). During a sabbatical year, 1977–1978, Gaston visited the laboratory of Professor Irvin C. Gunsalus at the University of Illinois Urbana-Champaign, where he became involved in studying cytochrome P450 [1,2]. This enzyme played a central role in his research in the following years. After receiving several promotions, in 1992, Gaston was appointed the INSERM Research Director (Directeur de Research, DR1). After his retirement in 2000, Gaston continued his scientific work as an Emeritus Director of Research in INSERM, Unité 779, Le Kremlin Bicêtre. 

The predominant part of Gaston’s scientific heritage is devoted to the effects of hydrostatic pressure on proteins and their use in the studies of protein conformational dynamics and function. His first publication in this field, “High-pressure spectrometry at sub-zero temperatures,” appeared in 1982 [3]. In this paper, Gaston and his co-authors describe the high-pressure optical cell of their construction and report the results of their pioneering study on the effects of pressure on the spin equilibrium of cytochrome P450. Since then, the idea of using pressure to displace protein conformational equilibria and perturb protein–solvent interactions became the keynote of Gaston’s research. Likewise, cytochrome P450 became the main object for Gaston’s studies for over 20 years. Gaston’s research on the effects of hydrostatic pressure on substrate binding, equilibria of heme iron ligation, and the protein–protein interactions of cytochromes P450 [4,5,6,7,8,9,10,11,12,13] has become a part of the golden fund of cytochrome P450 science and has served as a prototype for pressure perturbation studies in other laboratories. Another essential part of Gaston’s heritage is a series of studies on cytochrome c oxidase and its interactions with cytochrome c in collaboration with Jack Kornblatt [14,15,16]. During the most recent years, Gaston applied pressure perturbation to the studies of neuroglobin and other globins [17,18,19] and viroid RNA [20,21]. Regardless of the subject of Gaston’s research, his experimental approaches have always been distinguishable by their inventive design and innovative research strategies. The list of Gaston’s scientific publications includes over 150 experimental and review papers in reputable scientific journals. He attended various scientific congresses as a participant and keynote speaker (Figure 2).

Besides being a prominent scientist, Gaston was distinguished by his remarkable engineering skills. Since the experimental equipment that he needed for his innovative studies had not yet been (and remains not to be) commercially available, he designed and built research instruments by himself. The list of unique equipment built by Gaston in his laboratory in Paris includes thermostatically controlled high-pressure optical cells withstanding pressures up to 6 kbar, pressure jump and high-pressure stop-flow devices, and photoacoustic spectrophotometer. 

In the European Union Biotechnology Program projects, Gaston collaborated with many laboratories in Germany, France, and the United Kingdom. He also coordinated two multilateral research grants funded by the International Association for Cooperation with Scientists from the former Soviet Union (INTAS). The list of Gaston’s collaborators includes such scientists as Gregory A. Petsko (Wayne State University, Detroit, MI, USA), Jack and Judith Kornblatt (Concordia University Montreal, Quebec, QC, Canada); Stephen G. Sligar (University of Illinois, Urbana, IL, USA); Dmitri R. Davydov (Institute of Biomedical Chemistry, Moscow, Russia); Christiane Jung (Max Delbrück Centrum for Molecular Medicine, Berlin, Germany); and many more. 

Below we give the floor to some of Gaston’s collaborators and friends to allow them to share their memories about this remarkable scientist. 

**Gregory A. Petsko**

He was one of the nicest human beings I ever knew. He was also a brilliant experimentalist, with some of the best hands I have ever seen and a keen instinct for doing just the right experiment.

When you are a young scientist, it is impossible to overstate the importance of your intellectual growth and self-esteem that come about when an older scientist takes an interest in you, teaches you, and encourages your own ideas. During my time in Prof. Pierre Douzou’s lab as an EMBO fellow in 1973, I benefited from just such interest not only from Pierre but also from Gaston. I had been a protein crystallography graduate student, so much of what I know about studying proteins in solution, including enzyme kinetics, I learned at the bench from Gaston. He probably never had a more willing—and more inept—pupil. However, a more patient, generous, and able teacher one could not have asked for. 

We were trying to measure enzyme reactions in aqueous–organic media at subzero temperatures so that we could trap kinetically significant intermediates and characterize them spectroscopically [22]. My dream, which he shared, was that, ultimately, I could use these same techniques, pioneered by him and Pierre, to accumulate such intermediates in enzyme crystals so that their structures could be determined at atomic resolution. That dream was fully realized twenty-seven years later, when Steve Sligar, Dagmar Ringe, Ilme Schlichting, and I successfully determined the three-dimensional structures of every kinetically significant intermediate in the very enzyme Gaston had taught me about those many years before, cytochrome P450. 

The past never ceases to call to us, and if we heed that call, it can summon up bad memories as well as good ones. I have nothing but the best memories of my time in Paris, my time before striking out on my own as a scientist, and my time with Gaston Hui Bon Hoa. My life is so much better for having known him.

**Stephen G. Sligar**

In science, it is the interpersonal connections that define opportunities and career paths. When I was a graduate student in physics at the University of Illinois, wandering into the Biochemistry Department led me to meet I. C. Gunsalus or Gunny. A giant in microbiology and biochemistry, he was a close friend with Marianne Grunberg-Manago and Pierre Douzou at the Institute de Biologie Physico Chimique (IBPC). Thus, when I was a tenured professor, I sought Gunny’s advice as to where to spend a sabbatical year—and his suggestion was Paris, IPBC, and Pierre’s laboratory. 

Through some magic of these greats, I received a Fullbright Fellowship in 1989 to support the move of our family to Paris. After arriving, I learned my de jour mentor was a most energetic Gaston Hui Bon Hoa. What a great time scientifically and a most memorable personal life experience. Never, to this day, have I met anyone with such boundless energy and enthusiasm for doing experiments. Gaston would always prefer to do another experiment than write anything up for publication. His filing cabinets were full of so much data that, at any point, if one wanted a manuscript, you just went to the cabinet, pulled out a folder, and started writing. The IBPC was such a motivating place that we returned for several summers after the sabbatical. We formed partnerships with Jack and Judy Kornblatt, Dimitri Davydov, Christiane Jung, and others. This family spawned many discoveries. One memorable idea was hatched at a group wine event on the top floor of the IBPC. We were discussing water under pressure and then the osmotic forces that can act to desolvate. One problem facing molecular biologists was the “star” activity of restriction endonucleases. A list of solutions to avoid to prevent the loss of 6-base pair recognition was at the back of every catalog. After a lot of wine, it occurred to us that these were all osmolytes. Several pioneering papers ensued, showing that osmotic pressure indeed removed bridging hydrogen bonding water between protein and the outer base pairs that could be reversed by applying hydrostatic pressure! 

Gaston was unique and a source of motivation and companionship that is missed by all. He continued doing experiments after IBPC, working with Mike Marden, and thinking of new ways to advance biophysics through careful measurement. A real pioneer!

**Jack A. Kornblatt**

How do I remember our love affair with IBPC, Pierre Douzou, and most especially, with Gaston Hui Bon Hoa? It was in June 1982 when I and Judy, my wife, arrived in Paris from Cap d′Ail after a month at a French-language school. It was cold and raining heavily. We were wet and tired when we eventually came to our apartment and went back down to the bar on the rez de chaussee, where we tried to calm down with a large brandy each. A very inauspicious beginning to a wonderful year!

I went on up the street to IBPC and was introduced to Gaston Hui Bon Hoa, with whom I would work that year and during the summers in subsequent years. I had come to do low-temperature studies on the cytochrome c oxidase. However, Pierre explained that Gaston had just finished building a high-pressure optical cell (la bombe) that could be interfaced with a spectrophotometer or fluorometer. I put the oxidase into Gaston’s “la bombe” and was hooked. The world’s most exciting data poured out. The influence of high hydrostatic pressure on the oxidase was phenomenal. It allowed the catching and trapping of the intermediates with very large volume changes and helped us point out the energy transduction mechanism. All throughout this, Gaston guided my hands. 

Later, when Steve Sligar came into the lab, he brought the beautiful structure of cytochrome P450cam. After staring at it for days, it finally occurred to me that osmolytes worked by reducing the activity of water and that it might be one of the reasons why they aid camphor in gaining access to a water-filled pocket. This realization launched me to finally complete the description of the energy transduction and catalytic cycles of the oxidase. The papers wrote themselves. 

Very recently (2008), the team at Cornell developed high-pressure SAXS. Were Gaston with us today, he would be incredibly excited. “I have to try it out on P450cam”, he would have said. He was a man with boundless enthusiasm and energy, and he happily shared it. 

At the beginning of this overlong appreciation, I used the term love affair. It was just that. Additionally, by the way, 20 years later, I finally got to do the low-temperature work that I had initially planned!

**Christiane Jung**

During my research stay in the laboratory of Professor Irvin C. Gunsalus at the University of Illinois at Urbana-Champaign in 1982, I heard that there was a talented scientist in the INSERM laboratory of Professor Pierre Douzou at the Institute de Biology Physico-Chimique (IBPC) in Paris who was setting-up subzero-temperature and high-hydrostatic pressure equipment for studying the conformational behavior of cytochrome P450. His name was Gaston Hui Bon Hoa. I met Gaston personally for the first time in 1991 during my two-month stay as a visiting scientist in the laboratory of Professor Pierre Douzou. I was impressed by the enthusiasm with which Gaston built the experimental equipment. Our studies focused on cytochrome P450—the enzyme I had been working on since 1973 in Professor Klaus Ruckpaul’s group at the Academy of Sciences in Berlin and which remained the main object of studies in my own laboratory at the Max Delbrück Centrum for Molecular Medicine in Berlin later [11]. The high-pressure approach used by Gaston encouraged me to keep working together. During another seven-month stay in Gaston’s laboratory in 1993, we worked together with B. Canny and J.C. Chervin from the Université Pierre et Marie Curie Paris, Laboratoire de Physique des Milieux Condensés, to design a high-pressure cell with sapphire anvils, which we later used in Berlin for FTIR spectroscopic investigations on the carbon monoxide complex of cytochromes P450 [23]. Gaston visited my laboratory in Berlin several times as a participant in the EU project BIO2-942060. Looking back, I have to say that without Gaston, I would not have been able to establish high-pressure technology on my own.

In addition to being an inspiring scientist, Gaston was a good friend and, as I realized, a great family man. I fondly remember a trip with his family in 1991 to the Pont de Normandie near Le Havre and Honfleur, a beautiful little French port town in Normandy near the Seine estuary in the English Channel. In addition, I had the pleasure of attending his 80th birthday party in Paris seven years ago. I will always remember Gaston.

**Dmitri R. Davydov**

I first met Gaston Hui Bon Hoa at the international conference on Cytochromes P450 in Moscow in 1991. At that moment, the focus of my studies was (and remains to be) on the catalytic mechanisms and the protein–protein interactions of cytochromes P450. I knew very little about the effects of hydrostatic pressure on proteins and their use in biophysical studies. Still, I knew Gaston’s name from his publications on the impact of pressure on P450cam [7,24]. My attention was drawn to Gaston’s presentation at the meeting. It spurred my interest in the use of pressure perturbation. I got captivated by his enthusiasm and ideas on how pressure perturbation may be used in P450 research. Soon, I joined his research group in the laboratory of Pierre Douzou in IBPC in Paris as an INSERM fellow (“poste verte”). It was a wonderful time. I enjoyed the creative atmosphere in the lab and admired Gaston’s engineering ingenuity. Besides researching pressure-induced transitions in P450 2B4 (which I brought from my lab in Moscow) [5,25], I also participated in Gaston’s engineering efforts. I designed data acquisition and analysis software for high-pressure spectroscopy, which became a core of my SpectraLab software that I still use in my research. At the end of my fellowship, we were successful in acquiring an INSERM collaborative grant followed by an INTAS multilateral research grant. This funding allowed us to continue our collaboration [9,26,27]. From 1993 to 1999, I enjoyed visiting Gaston’s lab several times a year. After my move to the US, our collaboration continued [6], and in 2002, Gaston visited me in the University of Texas Medical Branch (UTMB, Galveston, TX) to install his high-pressure equipment, which I still use in my research. Later, we met several times in San Diego, Paris, and other places. Owing to my collaboration with Gaston, pressure perturbation became an integral part of my research strategy, and the effects of pressure on proteins became another focus of my scientific interests. I enjoyed our collaboration and friendship a lot. Besides being a talented experimentalist and prominent scientist, he was also a wonderful person and a good friend.

Collaboration with Gaston became momentous and life-defining for many of his colleagues and friends. Gaston’s ingenuity and enthusiasm swept them along with him and promoted the development of high-pressure bioscience all over the world. So, let this Special Issue become our tribute to this impassioned pathfinder, a talented scientist, and great friend. 

## Figures and Tables

**Figure 1:**
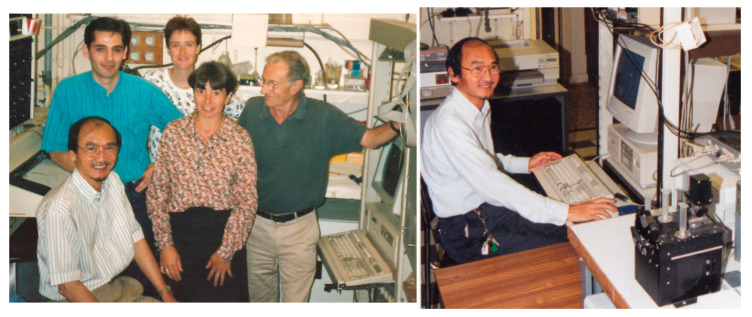
**Gaston Hui Bon Hoa at the Institute of Physico-Chemical Biology (Paris, France)**. **Left**: Laboratory of Prof. Pierre Douzou (INSERM Unité 310). Front row: Gaston Hui Bon Hoa, Pascale Debey, Pierre Douzou. Back row: Carmelo Di Primo, Sylvie Le Bihan. Summer 1992, Paris, France. **Right**: Gaston Hui Bon Hoa at his workplace, November 1996.

**Figure 2:**
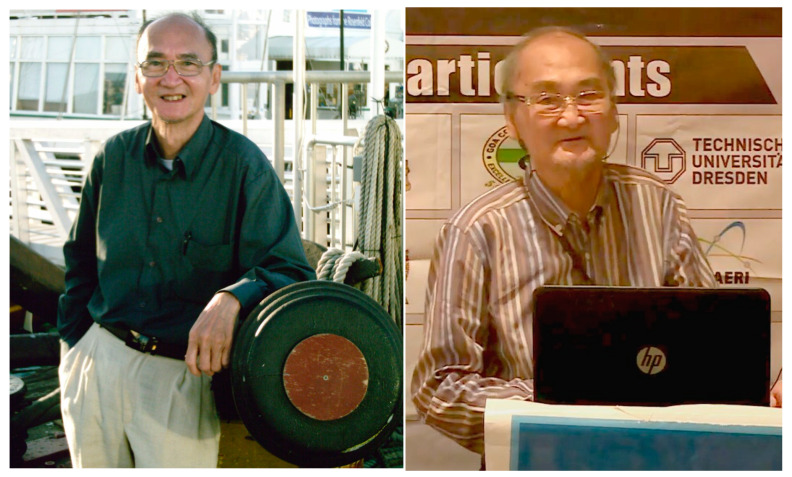
**Gaston Hui Bon Hoa at Scientific Meetings**. **Left**: A photo taken during the Fifth International Conference on High-Pressure Biosciences and Biotechnology (San Diego, CA, USA, September 2008). **Right**: Gaston presents his talk at the 7th Asia-Pacific Biotech Congress (Beijing, China, July 2015, Available online: https://www.youtube.com/watch?v=-4vs2mvqSGs (accessed on 18 July 2021)).

## Data Availability

Not applicable.
